# Study of Surface Structure Changes for Selected Ceramics Used in the CAD/CAM System on the Degree of Microbial Colonization,* In Vitro* Tests

**DOI:** 10.1155/2019/9130806

**Published:** 2019-06-12

**Authors:** Maciej Dobrzynski, Magdalena Pajaczkowska, Joanna Nowicka, Aleksander Jaworski, Piotr Kosior, Maria Szymonowicz, Piotr Kuropka, Zbigniew Rybak, Zdzislaw A. Bogucki, Jaroslaw Filipiak, Sara Targonska, Aneta Ciupa-Litwa, Anna Han, Rafal J. Wiglusz

**Affiliations:** ^1^Department of Conservative Dentistry and Pedodontics, Wroclaw Medical University, Krakowska 26, 50-425 Wroclaw, Poland; ^2^Department of Microbiology, Wroclaw Medical University, T. Chalubinskiego 4, 50-368 Wroclaw, Poland; ^3^Department of Experimental Surgery and Biomaterial Research, Wroclaw Medical University, Bujwida 44, 50-368 Wroclaw, Poland; ^4^Department of Histology and Embryology, Wroclaw University of Environmental and Life Sciences, Norwida 31, 50-375 Wroclaw, Poland; ^5^Department of Dental Prosthodontics, Wroclaw Medical University, Krakowska 26, 50-425 Wroclaw, Poland; ^6^Department of Biomedical Engineering, Mechatronics and Theory of Mechanisms, Faculty of Mechanical Engineering, Wroclaw University of Science and Technology, Poland; ^7^Institute of Low Temperature and Structure Research, Polish Academy of Sciences, Okolna 2, 50-422 Wroclaw, Poland

## Abstract

In the article has been presented an analysis of susceptibility of selected dental materials, made in the CAD/CAM technology. The morphology and structural properties of selected dental materials and their composites were determined by using XRPD (X-ray powder diffraction) techniques, as well as the IR (infrared) spectroscopy. Moreover, an adhesion as well as development of biofilm by oral microorganisms has been studied. It has been shown that a degree of the biofilm development on the tested dental materials depended on microorganism genus and species.* Streptococcus mutans* has demonstrated the best adhesion to the tested materials in comparison with* Candida albicans* and* Lactobacillus rhamnosus*. However, the sintered materials such as IPS e.max® and the polished IPS e.max® have showed the best “anti-adhesive properties” in relation to* S. mutans* and* L. rhamnosus* that have not formed the biofilm on the polished IPS e.max® sample. Furthermore,* S. mutans* have not formed the biofilm on both surfaces. On the contrary to* S. mutans* and* L. rhamnosus*,* C. albicans* has demonstrated the adhesive properties in relation to the above-mentioned surfaces. Moreover, in contrast to* S. mutans* and* C. albicans*,* L. rhamnosus* has not formed the biofilm on the polished IPS Empress material.

## 1. Introduction

In the modern restorative dentistry, the use of the CAD/CAM (computer-assisted design and manufacturing) technology is becoming more and more important in the preparation of the fillings that allow restoring a normal function of tooth tissues [1, 2]. This technology makes it possible to save time related to the cooperation between the dentist and the dental laboratory and the time spent on the preparation of highly aesthetic durable and functional dental restorations that are characterized by excellent anatomy and marginal sealing. Hybrid ceramic materials are particularly important among the currently used materials, and depending on the composition, there have been manifested different physical and biological properties. Taking into account the impact of the dental fillings use on “its surroundings” and the prospective duration of keeping it in the mouth as well as its susceptibility to plaque formation, it is one of the most important features.

Biofilm may contain about 1,000 species of bacteria [3]. It is formed by soft deposits adhering to both the surface of the teeth and prosthetic restorations. Its main “mass” consists of bacteria (60-70% of the volume of plaque) embedded in the amorphous matrix that is mainly composed of glycoproteins of saliva, glucans, and fructans of bacterial origin. The formation of the mature plaque is related to the natural production of the pellicle on the surface of teeth. The pellicle is mainly composed of glycoproteins, phosphoproteins, lipids, and bacterial adhesin receptors [4, 5]. Bacteria bind to the pellicle within the first 4 hours after cleaning the teeth.

The plaque can contain both Gram-positive and Gram-negative bacteria. Gram-positive bacteria, mainly granulomas (*Streptococcus mutans*,* S. salivarius*,* S. motis,* and* Lactobacillus spp.*), dominate in the supragingival plaque, whereas anaerobic Gram-negative ones (*Actinobacillus spp.*,* Campylobacter spp.*,* Fusobacterium nucleatum*,* Porphyromonas gingivalis*) dominate in the subgingival plaque [6]. It is worth mentioning that as the plaque becomes thicker, the access of oxygen to its deeper layers decreases, which in turn results in the increasing amount of anaerobic bacteria growing there [5].

The occurrence of tooth decay is mostly associated with the supragingival biofilm whereas the occurrence of periodontal and gum diseases is associated with the subgingival plaque [7, 8]. Microorganisms that make up the plaque, such as* Streptococcus mutans* and* Streptococcus sobrinus,* are considered to be the original pathogens in the etiology of tooth and cementum decay, whereas the strains of* Lactobacillus* are responsible for the progress of the disease [3].

Fungi, especially yeast-like fungi, play a huge role in oral infections related to the formation of the biofilm.* Candida albicans* mainly participates in the development of the biofilm that is associated with the extensive use of the prosthetic restorations. Acrylic material is the main fillings and according to the literature* C. albicans* has strong adhesive properties to this type of surfaces [9]. The type and nature of the material surface are also important in terms of the adhesion of microorganisms. A number of authors showed that surface roughness and free energy as well as hydrophilic or hydrophobic properties are of great importance [10]. The surface roughness increases the adhesion of microorganisms and the formation of the biofilm. Dentists strive to use durable and aesthetic materials in the dentistry. The materials with antimicrobial activity as well as inhibiting the adhesion and colonisation of microorganisms in the mouth are still searched. Therefore the aim of this study was to compare adhesion as well as development of the biofilm by chosen oral microorganisms on the ceramics materials in regard to their roughness.

## 2. Materials and Methods

### 2.1. Materials

The study was carried out in relation to 4 materials from the group of hybrid ceramics used to create fillings in the CAD/CAM technology, which were divided into 2 groups: non-polished materials (Vita Enamic, IPS Empress Multi, IPS Empress) and polished materials (polished Vita Enamic, polished IPS Empress Multi, polished IPS Empress).

**Figure 1:**
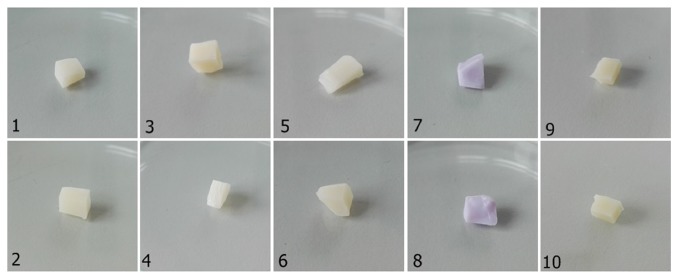
Dental material used in the study: 1: Vita Enamic; 2: Vita Enamic - polished; 3: IPS Empress Multi; 4: IPS Empress Multi - polished; 5: IPS Empress; 6: IPS Empress - polished; 7: IPS e.max® before sintering; 8: IPS e.max® - polished before sintering; 9: IPS e.max® - after sintering; 10: IPS e.max® - polished after sintering.

The study also included samples of IPS e.max® before sintering (IPS e.max® before sintering, polished IPS e.max® before sintering) and after sintering (IPS e.max® after sintering, polished IPS e.max® after sintering) (Table 1, Figure 1). The materials samples were sterilized in a Type B autoclave in accordance with DIN EN 13060-2 (temperature=134°C, time=30 min, pressure= 2,2 bar).

### 2.2. Structural Analysis

Five materials were used in this study, IPS e-max CAD (Ivoclar Vivadent, Schaan Liechtenstein), IPS e-max sintered at 800°C, IPS Empress CAD (Ivoclar Vivadent, Schaan Liechtenstein), IPS Empress Multi CAD (Ivoclar Vivadent, Schaan Liechtenstein), and Vita Enamic (VITA Zahnfabrik, Bad Säckingen, Germany). Chosen materials represent the most common used group in CAD/CAM technic. CAD/CAM blocks was cut into 1mm slice and polished on a wet rotary polisher with 800 and 1200-grit silicon paper.

Powder X-ray diffraction patterns were measured in 2*θ* range of 5-80° 2Θ by using a PANalytical X'Pert Pro X-ray diffractometer equipped with Ni-filtered Cu K*α*1 radiation (K*α*1 = 1.54060 Å, U = 40 kV, I = 30 mA). The experimental XRD patterns were correlated with the patterns obtained from database of inorganic crystal structure (COD) and analyzed. Ceramics phase was identify using Match! 3.6.1.115 software.

The ATR (Attenuated Total Reflection) spectra were measured using Nicolet iS50 FT-IR (Thermo Scientific) spectrometer equipped with an Automated Beamsplitter exchange system (iS50 ABX containing DLaTGS KBr detector), built-in all reflective diamond ATR module (iS50 ATR), Thermo Scientific PolarisTM and HeNe laser as an IR radiation source. Spectral resolution was set to 4 cm^−1^.

### 2.3. Measurement Values of Contact Angles

**Figure 2:**
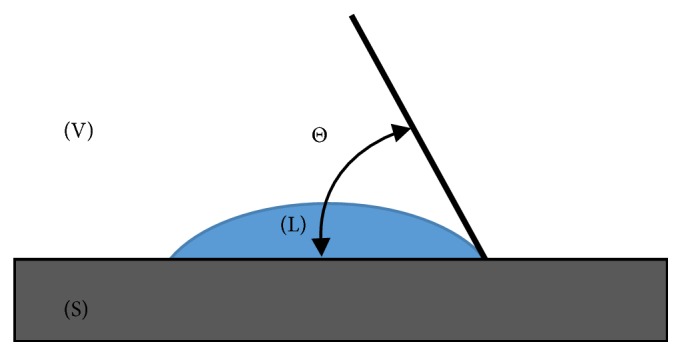
A scheme for determining the contact angle Θ of a solid surface.

Wettability is the ability of a liquid to maintain contact with a solid surface, and it is controlled by the balance between the intermolecular interactions of adhesive type (liquid to surface) and cohesive type (liquid to liquid). Wettability of the tested materials surface was determined by the method of drop deposition. In this method, the wettability is determined on the basis of the measuring liquid drop shape placed on the surface to be tested. The measure of wettability is the contact angle Θ between the tangent to the surface of the drop and the surface of the solid. The tangent has its beginning at the point of contact of the three phases: constant (S), liquid (L), and gas (V), Figure 2.

The tests were carried out using a goniometer Surftens Universal (OEG GmbH, Germany). Distilled water was used as the measuring liquid and the average drop volume was 0.5 ml. The air temperature in the measurement space was 22°C, and the humidity was 41%. Before applying the measuring drop, the surface of the sample was degreased and leveled on the goniometer table. Five measurements were taken for each of the tested samples.

### 2.4. Quantitative Assessment of Adhesive Properties and the Ability to Form a Biofilm

**Figure 3:**
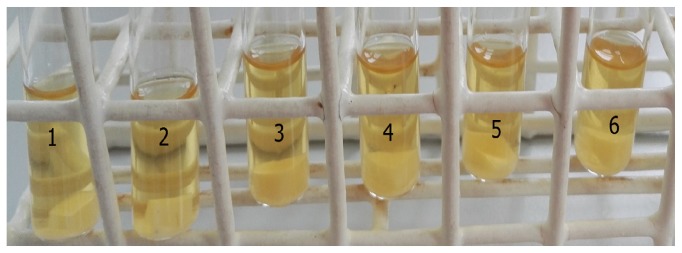
Samples of dental materials in the suspension of the strain of S. mutans. (sample photo) 1: Vita Enamic; 2: polished Vita Enamic; 3: IPS Empress Multi; 4: polished IPS Empress Multi; 5: IPS Empress; 6: polished IPS Empress.

A suspension of microorganisms corresponding to the density of 0.5 on the McFarland scale in case of fungi (1,5x10^6^ CFU/ml) and 1.0 in case of bacteria (3,0x10^8^ CFU/ml) was prepared on the basis of fresh cultures of analysed strains. To obtain the suspension of strains of the appropriate density, liquid media Sabouraud Dextrose Broth (Biocorp), Brain Heart Infusion Broth, BHI (Biocorp), and Man-Rogosa-Sharpe Broth, MRS (Biocorp), were used for* C. albicans*,* S. mutans,* and* L. rhamnosus*, accordingly (Figure 3). The sterile dental materials were put into the prepared suspension of microorganisms. After the incubation period of 48 hours at the temperature of 37°C (*S. mutans*: under microaerophilic conditions (desiccator);* L. rhamnosus*: under anaerobic conditions (Genbag anaer system);* C. albicans*: under aerobic conditions), the materials were rinsed three times in NaCl and shaken in 1ml of 0.5% of the saponin solution (Sigma) for 1 minute. The obtained suspension of microorganisms, desorbed from the material surface, was cultured quantitatively on solid media, and after the incubation period, colonies formed were counted and the number of colony-forming units per 1 ml of the suspension (cfu/mL) was assessed. This procedure was repeated three times for each sample.

The samples were cut of blocks supplied by the manufacturer using fine-grit diamond dental drills (NTI-Kahla GmbH) mounted on a dental handpiece (NSK S-Max M600 L) with the maximum speed of 100,000 rpm. The drills used corresponded to the drills used for the processing of fillings that are cemented in the patient's mouth. Samples were polished with the use of ceramic polishers (Kenda Dental Polishers) mounted on a low-speed handpiece (NSK S-Max M 25 l) with the maximum speed of 15,000 rpm and water spray cooling.

In all cases, drills of the same shape and grit, produced by the same manufacturer, were used to machine the surfaces of the samples. The machining process was carried out with the use of diamond drills and NSK turbine rotor working within the range of 400,000 rpm.

The following standard strains were used in the study:* Streptococcus mutans* (ATCC 25175),* Candida albicans* (ATCC 90028), and* Lactobacillus rhamnosus* (ATCC 9595).

### 2.5. Morphometric Analysis

After the incubation, the material was stained with the solution of acridine orange (Invitrogen TM) and Rhodamine B (Invitrogen TM) and then analysed under the fluorescence microscope Nikon Eclipse 80i using the UV-2A (EX- 330-380, DM-400, BA-420) and G-2A filter (EX -510-560, DM-575, BA 590).

### 2.6. Statistical Analysis

Data were analyzed using Analysis Tool Pack for MS Excel (Microsoft Corporation, Redmond, Washington, United States). Data were checked by Kolmogorov-Smirnov and Shapiro-Wilk tests. To compare between the groups, ANOVA test was performed. The significance level was set at p ≤ 0.05.

## 3. Results

### 3.1. X-Ray Diffraction

**Figure 4:**
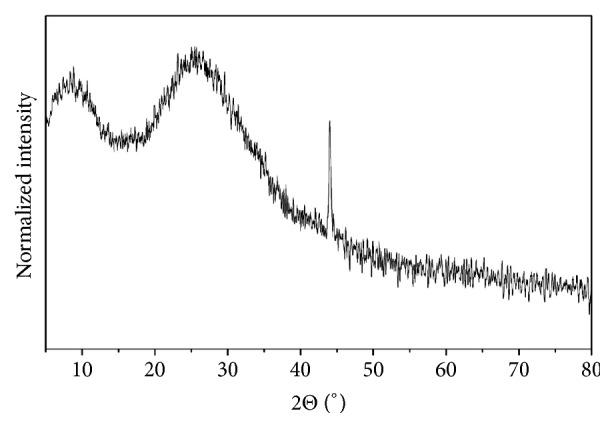
X-ray powder diffraction patterns of the Vita Enamic.

Ceramic materials used for prosthetic restorations are mostly composites of acrylic and methacrylate polymers filled with a ceramic phase. X-ray diffraction was carried out to determine the crystalline phases in the used materials. The result showing the based inorganic compounds in CAD/CAM materials and the narrow shape of the peaks suggest the domination of the crystalline phase. Only Vita Enamic materials cannot be clearly characterized by this method, and X-ray diffractogram is presented on Figure 4. High background in range of low angles can be associated with a significant part of the non-crystal phase. As it has been written on the producer's website, the composition contains 14% of a weight in polymer phase, poly (urethane dimethacrylate-co- ethylene glycol dimethacrylate) [17]. High intensity of x-ray diffraction at this range can cover another peaks associated with inorganic component. One sharp peak at 44° is observed and can be related to SiO_2_, Al_2_O_3_, or ZrO_2_, which are part of inorganic phase [17].

**Figure 5:**
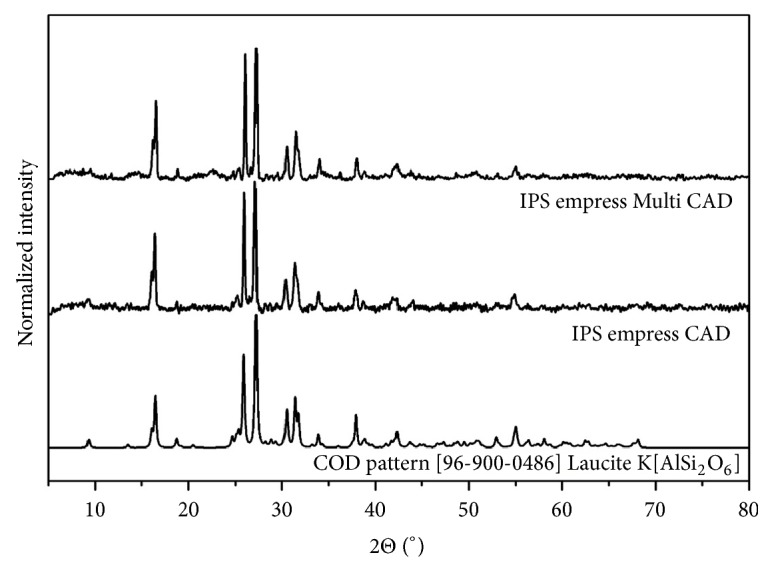
X-ray powder diffraction of the samples IPS Empress CAD and IPS Empress Multi CAD correlated with COD pattern.

**Figure 6:**
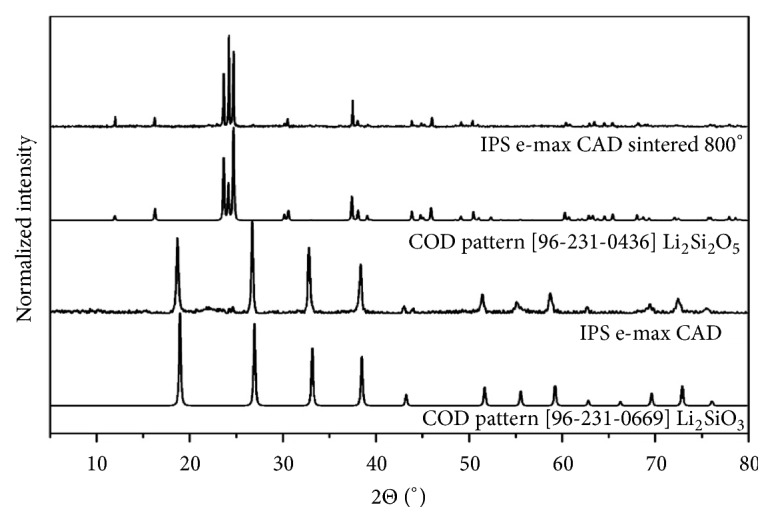
X-ray powder diffraction of the samples IPS e-max CAD and IPS e-max CAD sintered at 800°C correlated with COD pattern.

Characteristic peak lines observed for the IPS Empress and IPS Empress Multi (Figure 5) clearly correspond to K[AlSi_2_O_6_], referents pattern by COD (Crystallography Open Database) no. 96-900-0486. Prime peaks refer to lithium silicate crystals (Li_2_SiO_3_), COD no. 96-231-0669, found for IPS e-max CAD and lithium disilicate (Li_2_Si_2_O_5_), COD no. 96-231-0436, for IPS e-max CAD sintered at 800°C (Figure 6). Changes in the composition occurring during the sintering process could be observed in the alteration of the material color. The initial purple hue changes to a color similar to the natural of the teeth.

### 3.2. Infrared Analysis

**Figure 7:**
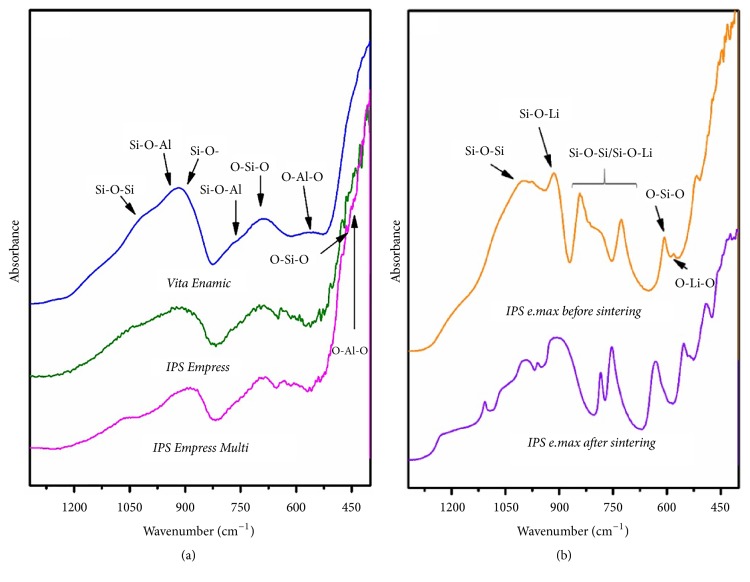
IR-ATR spectra of the studied materials: (a) hybrid blend and Leucite glass ceramics as well as (b) lithium disilicate glass ceramics.

The IR spectra of the studied materials are shown in Figure 7. Analysis of phonon properties of the samples requires the knowledge of their structure. Three ions can be distinguished in the structure of glass ceramic: network forming, intermediate and modifying [18, 19]. The Si^4+^ ions form glass network due to their ability to build up silicon-oxygen bridges and thus 3D network. One of the most important intermediate ions is aluminium. The Al^3+^ cations can be coordinated by four or six oxygen atoms creating AlO_4_/AlO_6_ units. Four-coordinated aluminium (together with silica ions) is involved in formation of the glass structure, whereas six-coordinated aluminium breaks it. The introducing of modifying ions (mainly alkali cations) produces the broken silicon-oxygen bridges (Si-O-) [20].

The main component of the samples is SiO_2_ oxide; thus, the previously reported vibrational studies of silica and silicate glass can be used as the reference in the interpretation of infrared spectra of our glass ceramics. According to the literature, amorphous silica shows strong IR bands at around 470 with a shoulder at 570 (bending O-Si-O), 800 (symmetric stretching Si-O-Si), 1100-1200 (asymmetric stretching Si-O-Si), and 3470 cm^−1^ (stretching Si-OH) [21, 22]. Any incorporation of metal cations into the structure of SiO_2_ glass results in the modifying of its properties (e.g., distances between atoms) that are reflected in the infrared spectra. Ferraro et al. investigated the structure of lithium and sodium silicate glasses by vibrational spectroscopy [23]. It was demonstrated that, for example, the frequency of the ~1100 cm^−1^ band (stretching Si-O-Si) decreases with an increase of the alkali content in both glass types. This observation clearly indicates that the Si-O vibrational bond force constants are weakened as level of K_2_O/Na_2_O dopant is higher [23]. As was mentioned above, the introducing of modifying ions (e.g., Na^+^, K^+^, Ca^2+^) results in appearance of broken Si-O- bridges. The position of IR bands associated with vibrations of silicate tetrahedral with non-bridging oxygen atoms (NBO atoms) is strongly dependent on the number of NBO atoms [22]. For instance, vibrational modes at around 1200 and 850 cm^−1^ are associated with [SiO4]^4-^ with 0 and 4 NBO atoms, respectively [24].

IR spectra of Vita Enamic, IPS Empress, and IPS Empress Multi samples are very similar (Figure 7(a)). They are characterized by broad bands at around 1150-900, 770-600, and below 450 cm^−1^. Bands observed at the highest wave numbers (1135, 1013, 918 cm^−1^ for Vita Enamic, 1058, 926, 894 cm^−1^ for IPS Empress, and 1049, 960, 896 cm^−1^ for IPS Empress Multi) are associated with stretching vibrations of Si-O-Si, Si-O-Al, and Si-O- bridges. For comparison, infrared study of glass-ceramic materials from SiO_2_-Al_2_O_3_-Na_2_O-K_2_O-CaO system modified by variable molar ratio of SiO_2_/Al_2_O_3_ showed these bands in the 1180-903 cm^−1^ spectral range [20]. Bands localized at ~760 cm^−1^ correspond to the bending modes of Si-O-Si and Si-O-Al units. Two bands at around 690 and 600 cm^−1^ are associated with the oscillations of silico- and alumino-silico-oxygen rings [25, 26], while bending vibrations of O-Si-O and O-Al-O units give rise to maximum below 450 cm^−1^. Spectra of IPS e.max® (before and after sintering) samples are different from those described above (Figure 7(b)). There are several relatively sharp, mostly narrow, and well resolved bands. Before we discuss the origin of the disparity in the form of measured spectra, we assign the observed bands to vibrations of individuals structural elements in IPS e.max® samples. According to the specification given in Table 1 (composition and properties of dental materials), this dental material is composed of lithium disilicate glass ceramics. However, results of XRD measurements (see Figure 6) showed that the structures of IPS e.max® before and after sintering are different. The sintered and not sintered materials are composed of Li_2_SiO_3_ and Li_2_Si_2_O_2_, respectively. Thus, there are some disagreements between spectra of both samples (Figure 7(b)). The peaks localized at 1187, 1055, and 997 cm^−1^ (1221, 1107, 1045, and 993 cm^−1^) for not sintered (sintered) material are attributed to asymmetric stretching vibrations of Si-O-Si/Si-O-Li bridges. Their symmetric counterpart can be found at 970-960 cm^−1^. The bands at 914 and 840 cm^−1^ for not sintered sample (914 and 882 cm^−1^ for sintered sample) are known to be caused by vibrations of silicate tetrahedral with the NBO atoms. Bending modes of Si-O-Si/Si-O-Li units give rise to bands at 700-800 cm^−1^, whereas the oscillations of silico- and silico-lithium-oxygen rings can be found at 605 cm^−1^ (sample before sintering) and 630 cm^−1^ (sample after sintering). Bands below 600 cm^−1^ are associated with bending modes of O-Si-O and O-Li-O- bridges [27].

Furthermore, the IR spectra of Vita Enamic, IPS Empress, and IPS Empress Multi samples (presented in Figure 7(a)) are characterized by broad, not resolved bands with widths up to dozens wave numbers. Such observations are common for amorphous materials, which do not have long-range ordering. There is no such aspect at spectra of IPS e.max® samples. These results indicate that, in Vita Enamic, IPS Empress, and IPS Empress Multi materials, there is a higher percentage of silica oxide than in IPS e.max®.

### 3.3. Contact Angles

Table 2 presents the mean values of the contact angle Θ and the standard deviation (SD) for the samples tested. Five different materials were tested. Each of them appeared in two variants: sample 1 and sample 2. In the case of Vita Enamic material, sample 2 is characterized by a contact angle lower by 18% than sample 1. The observed difference is statistically significant (p < 0,05). In the case of materials, IPS Empress Multi, sintered IPS e-max, and IPS e-max, also differences between samples 1 and 2 were observed, but this time lower values of the contact angle are shown in sample 1. These are significant differences ranging from 32% to 35% (p < 0,05). The same is true for the material IPS Empress Multi, but the difference between samples 1 and 2 (9.3%) is not statistically significant. It should also be noted that coated materials show better wettability (lower value of the contact angle Θ) with respect to the non-surface modified material (Vita Enamic).

### 3.4. Microbial Adhesion

**Figure 8:**
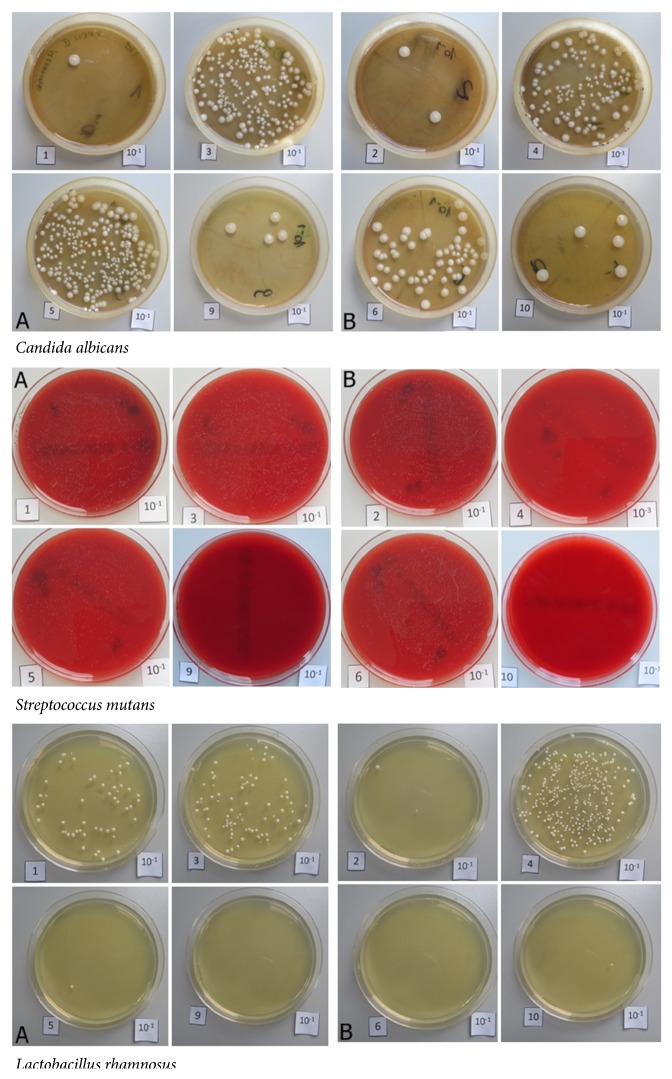
Macroscopic image of the colony of fungi, streptococci, and bacilli desorbed from the surface of dental materials under the influence of saponin. A: non-polished material; B: polished material.

**Figure 9:**
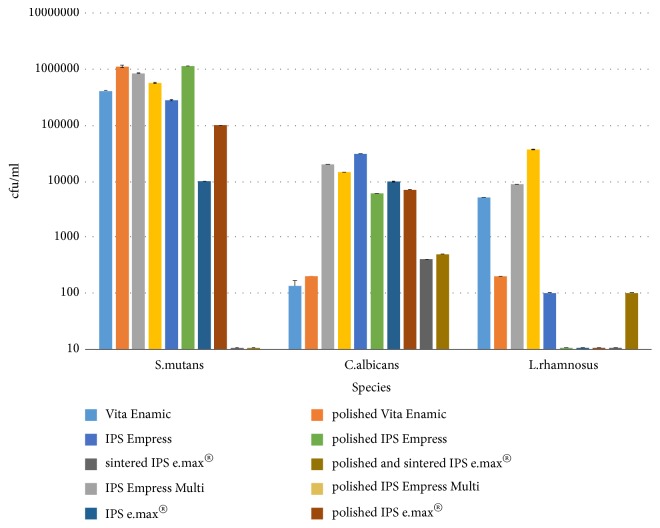
Quantitative assessment of adhesive properties of the analysed strains in relation to the selected dental materials. All the data in the groups were significantly different in statistical analysis at p≤0.05.

During the analysis of the susceptibility of the surface of dental materials to adhesion of Candida albicans, it was observed that non-polished surfaces showed a slightly higher susceptibility to adhesion of fungi, except for Vita Enamic. In the study, it was observed that Vita Enamic and polished Vita Enamic are the materials that were characterized by the lowest susceptibility to adhesion of* Candida albicans*. This microorganism demonstrated the highest adhesive properties in relation to the surfaces of the IPS Empress and IPS Empress Multi samples. Fungal cells were shown the approximately 100-fold higher adhesion to such surfaces compared to the analysed dental materials (e.g., Vita Enamic, polished Vita Enamic, IPS e.max® after sintering, or polished IPS e.max® after sintering). The highest adhesion in* S. mutans* was noted in polished Vita Enamic and polished IPS Empress groups, whereas* L. rhamnosus *was noted in IPS Empress Multi ceramics. Both* S. mutans and L. rhamnosus* did not adhere to the sintered IPS e.max®. The differences in the group of dental materials were statistically significant.  Figure 8 presents the macroscopic image of the colony of the analysed microorganisms desorbed from the surface. Table 2 and Figure 9 show the quantitative assessment of adhesive properties of strains in relation to dental ceramics.

**Figure 10:**
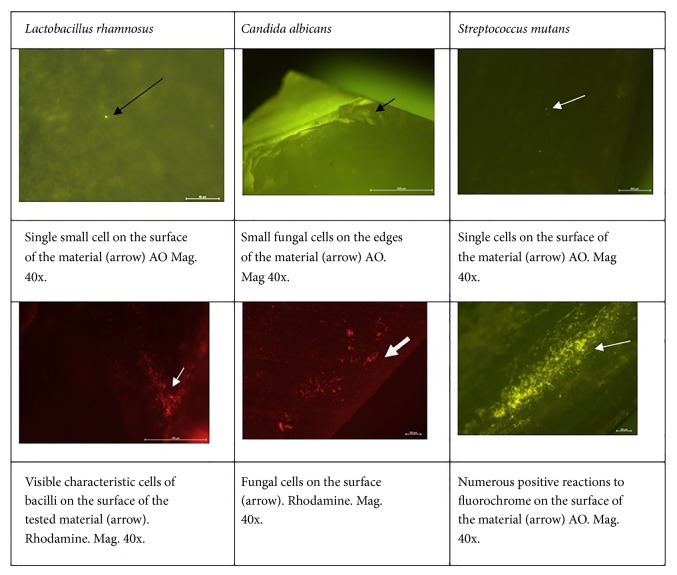
Image of cells on the surface of the tested materials after the use of rhodamine B and acridine orange (AO) obtained from the fluorescent microscope.

The morphological analysis confirmed the observations made during the bacteriological test (CFU/mL values). The materials demonstrated different adhesion and development of microorganisms on their surfaces (Table 3). In several cases, the highest cell growth and adhesion were observed on uneven, rough surfaces and sharp edges. The test show that the tested materials do not inhibit the development of microorganisms, but they only limit their adhesion to surfaces (please see Figure 10).

## 4. Discussion

The use of prosthetic fillings in dental restoration, such as inlays, onlays, veneers, or crowns, is necessary in case of a significant loss of hard tissues of teeth [28, 29]. Such restorations should be not only aesthetic, but also durable, functional, and hygienic. Hybrid ceramic materials are most frequently used for this type of restorations in the CAD/CAM technology; they ensure easy rehabilitation of lost hard tissues in case of their significant deficiency [30].

They have different physicochemical and mechanical properties such as flexibility and polishability, which affects their durability, aesthetics, and functionality. One of the most important characteristics of the filling is its susceptibility to plaque formation on its surface [31, 32]. It was proven that the surface type and nature are important in relation to the adhesion and colonisation of a given surface by microorganisms [9]. Attempts to develop new biocompatible materials that are not susceptible to adhesion of microorganisms and that enable full biointegration are made in dental restoration, dental surgery, and orthopaedics. Moreover, to obtain the best results, the surface of biomaterials is modified by tarnishing or roughing it with the use of various physical and chemical techniques or by applying bioceramic or hydroxyapatite coatings [33].

The analysis of the susceptibility of the selected dental materials to adhesion and formation of the biofilm by microorganisms in the mouth carried out as part of the study showed that non-polished surfaces were more susceptible to adhesion of microorganisms. Differences in the adhesion of microorganisms to the surface of polished and non-polished materials were statistically significant. Similar results have also been obtained by other authors [34–37]. Due to the dynamically developing materials science and state-of-the-art laboratory technologies, ceramic materials that are characterised by biocompatibility, high chemical resistance, low susceptibility to plaque formation, and low porosity are widely and successfully used in dental restoration [38, 39].

Materials from the group of dental ceramics consist of both glassy and crystalline phases. The glassy phase determines the aesthetics of ceramics, whereas the crystalline phase is responsible for the mechanical properties of the material. The degree of smoothness of ceramic materials is also important due to the fact that higher roughness and microscopic irregularities make them susceptible to the biofilm adhesion and growth [13].

If oral bacteria colonise the surface of the materials used as a filling or restoration, then over time they may become an etiological factor for caries, etc. The adhesion of the biofilm to abiotic surfaces and its formation by bacteria and fungi are important virulence factors of pathogens.

The adhesion of* Streptococcus mutans* strains to the surface of the tested materials was high regardless of the polishing method. It is probably related to the hydrophilicity of their surfaces. Similar conclusions were drawn by Quirynen et al. [40] in their study of the adhesion of* Streptococcus mutans* bacteria in the group of materials characterised by different degrees of hydrophilicity.

The roughness of the surface of materials placed in the mouth is their another important property which affects the initial adhesion and retention of microorganisms. H. Rashid [41] showed that the initial adhesion and colonisation of microorganisms on the tooth surface started in the areas with surface irregularities such as cracks, grooves, or defects. The colonisation of bacteria begins there and moves to other areas in the mouth. The formation of the biofilm on dental restoration surfaces may seem harmless, but bacteria colonising ceramic fillings may lead to secondary caries, changes in the oral mucosa, and the enamel demineralisation [31, 42]

The aim of this study was to determine the significance of the impact of the complete process of polishing ceramic materials on the degree of their roughness, and thus on the susceptibility of surfaces to adhesion of selected oral microorganisms. This may mean that in clinical conditions, despite the incomplete process of polishing surfaces of ceramic restorations, the colonisation of microorganisms in these areas increases. Therefore, it seems necessary to carry out additional studies in the group of patients with temporarily cemented ceramic fillings made in the CAD/CAM technology.

The smallest degree of colonisation was observed in case of samples subjected to sintering and polishing. This is due to the fact that in case of these samples, as a result of heat treatment and machining, we obtained the smoothest surfaces out of all the tested materials because surfaces are polished during the sintering phase [43].

After putting the ceramic restoration in place, it is often necessary to carry out an occlusal correction in the patient's mouth with the use of fine diamond drill bits and polishers for ceramic surfaces [44]. Due to the fact that such modified surfaces are characterised by higher roughness than glazed surfaces, it seems essential to conduct research on the susceptibility of such modified surfaces to colonisation of microorganisms [45].

## 5. Conclusions

Based on the conducted study and obtained results, it was stated that different degrees of the formation of biofilm on the tested dental hybrid ceramics can be observed depending on the species of microorganisms. Moreover,* Streptococcus mutans* demonstrated by far the best adhesion to the tested materials in comparison with* Candida albicans* and* Lactobacillus rhamnosus*. The sintered materials such as IPS e.max® and polished IPS e.max® showed the best “anti-adhesive properties” in relation to* Streptococcus mutans* and bacilli.* L. rhamnosus *did not form a biofilm on the polished IPS e.max® sample whereas* S. mutans* did not form a biofilm on both surfaces. Unlike granulomas and bacilli,* C. albicans* demonstrated adhesive properties in relation to the above-mentioned surfaces. Furthermore, unlike* S. mutans* and* C. albicans*,* L. rhamnosus* did not form a biofilm on the polished IPS Empress material.

## Figures and Tables

**Table 1:** Composition and properties of dental materials.

Material	Manufacturer	Composition and properties
IPS e.max®	Ivoclar Vivadent, Liechtenstein	lithium disilicate glass ceramics SiO_2_, Li_2_O, K_2_O, P_2_O_5_, SiO_2_, ZnO [11, 12]

Vita Enamic	Vita Zahnfabrik, Bad Säckingen, Germany	hybrid blend - consists of 86% of ceramics and 14% of polymer network fibres penetrating it [13]

IPS Empress	Ivoclar Vivadent, Liechtenstein	Leucite glass ceramics SiO_2_, Al_2_ O_3_, K_2_O, Na_2_O, dye/pigment [11, 12, 14, 15]

IPS Empress Multi	Ivoclar Vivadent, Liechtenstein	Leucite-reinforced glass ceramics [16]

**Table 2:** The mean values of the contact angle Θ and standard deviation SD.

Material	Sample	Contact angle Θ [^0^]	SD [^0^]
Vita Enamic	1	53.58	1.61
	2	43.94	1.23

IPS Empress	1	7.48	1.36

IPS Empress Multi	1	32.58	2.46
	2	35.94	1.99

IPS e-max after sintering	1	30.52	3.81
	2	45.02	2.44

IPS e-max before sintering	1	31.18	1.81
	2	47.56	2.11

**Table 3:** Number of colony-forming units per millilitre of the suspension (cfu/mL).

No.	Dental material	*C. albicans*	*S. mutans*	*L. rhamnosus*
		cfu/mL	cfu/mL	cfu/mL
1	Vita Enamic	1.3x10^2^	4.1x10^5^	5.1x10^3^

2	polished Vita Enamic	2.0x10^2^	1.1x10^6^	2.0 x10^2^

3	IPS Empress Multi	2.0x10^4^	8.5x10^5^	8.7x10^3^

4	polished IPS Empress Multi	1.45x10^4^	5.7x10^5^	3.7x10^4^

5	IPS Empress	3.07x10^4^	2.8x10^5^	1.0x10^2^

6	polished IPS Empress	6.0x10^3^	1.1x10^6^	0

7	IPS e.max®	9.8x10^3^	1.0x10^4^	0

8	polished IPS e.max®	7.1x10^3^	1.0x10^5^	0

9	sintered IPS e.max®	3.99x10^2^	0	0

10	polished and sintered IPS e.max®	4.99x10^2^	0	1.0x10^2^

## Data Availability

The data used to support the findings of this study are included within the article.

## References

[1] Lebon N., Tapie L., Duret F., Attal J.-P. (2016). Understanding Dental CAD/CAM for Restorations-Dental Milling Machines from a Mechanical Engineering Viewpoint. Part B: Labside Milling Machines. *International Journal of Computerized Dentistry*.

[2] Alghazzawi T. F. (2016). Advancements in CAD/CAM technology: Options for practical implementation. *Journal of Prosthodontic Research*.

[3] D'Argenio V., Salvatore F. (2015). The role of the gut microbiome in the healthy adult status. *Clinica Chimica Acta*.

[4] Teughels W., Van Assche N., Sliepen I., Quirynen M. (2006). Effect of material characteristics and/or surface topography on biofilm development. *Clinical Oral Implants Research*.

[5] Gopikrishna V. (2015). *Preclinical Manual of Conservative Dentistry and Endodontics*.

[6] Marsh P. D. (2010). Microbiology of dental plaque biofilms and their role in oral health and caries. *Dental Clinics of North America*.

[7] Li Y.-H., Hanna M. N., Svensäter G., Ellen R. P., Cvitkovitch D. G. (2001). Cell density modulates acid adaptation in Streptococcus mutans: Implications for survival in biofilms. *Journal of Bacteriology*.

[8] Seneviratne C. J., Zhang C. F., Samaranayake L. P. (2011). Dental plaque biofilm in oral health and disedase. *Chinese Journal of Dental Research*.

[9] McEldowney S., Fletcher M. (1987). Adhesion of bacteria from mixed cell suspension to solid surfaces. *Archives of Microbiology*.

[10] Monteiro D. R., Arias L. S., Fernandes R. A. (2017). Role of tyrosol on Candida albicans, Candida glabrata and Streptococcus mutans biofilms developed on different surfaces. *American Journal of Dentistry*.

[11] Nogueira A. D., Della Bona A. (2013). The effect of a coupling medium on color and translucency of CAD-CAM ceramics. *Journal of Dentistry*.

[12] Soygun K., Varol O., Ozer A., Bolayir G. (2017). Investigations on the effects of mouthrinses on the colour stability and surface roughness of different dental bioceramics. *The Journal of Advanced Prosthodontics*.

[13] Kim K. H., Loch C., Waddell J. N., Tompkins G., Schwass D. (2017). Surface characteristics and biofilm development on selected dental ceramic materials. *International Journal of Dentistry*.

[14] Sugiyama T., Kameyama A., Enokuchi T. (2017). Effect of professional dental prophylaxis on the surface gloss and roughness of CAD/CAM restorative materials. *Journal of Clinical and Experimental Dentistry*.

[15] Chen Y. M., Smales R. J., Yip K. H. K., Sung W. J. (2008). Translucency and biaxial flexural strength of four ceramic core materials. *Dental Materials*.

[16] Sedda M., Vichi A., Siena F. D., Louca C., Ferrari M. (2014). Flexural resistance of Cerec CAD/CAM system ceramic blocks. Part 2: Outsourcing materials. *American Journal of Dentistry*.

[17] VITA ENAMIC® - redefines load capacity https://www.vita-zahnfabrik.com/en/VITA-ENAMIC-24970,27568.html, 2018

[18] Li N., Ching W. Y. (2014). Structural, electronic and optical properties of a large random network model of amorphous SiO2glass. *Journal of Non-Crystalline Solids*.

[19] Jiang Z. H., Zhang Q. Y. (2014). The structure of glass: a phase equilibrium diagram approach. *Progress in Materials Science*.

[20] Partyka J., Leśniak M., Raman M. (2016). Infrared Spectroscopy Study on Structure and Microstructure of Glass-Ceramic Materials from SiO_2_-Al_2_O_3_-Na_2_O-K_2_O-CaO System Modified by Variable Molar Ratio of SiO2/Al2O. *Spectrochimica Acta Part A: Molecular and Biomolecular Spectroscopy*.

[21] Musić S., Živko-Babić J., Mehulić K. (1997). Microstructural Properties of Leucite-type Glass-ceramics for Dental Use. *Croatica Chemica Acta*.

[22] Musić S., Filipović-Vinceković N., Sekovanić L. (2011). Precipitation of Amorphous SiO2 Particules and Their Properties. *Brazilian Journal of Chemical Engineering*.

[23] Ferraro J. R., Manghnani M. H., Basile L. J. (1973). Infrared absorption spectra of lithium and potassium silicate glasses at high pressure. *Journal of Applied Physics*.

[24] Merzbacher C. I., White W. B. (1991). The structure of alkaline earth aluminosilicate glasses as determined by vibrational spectroscopy. *Journal of Non-Crystalline Solids*.

[25] Sitarz M., Mozgawa W., Handke M. (1999). Rings in the structure of silicate glasses. *Journal of Molecular Structure*.

[26] Handke M., Sitarz M., Mozgawa W. (1998). Model of silicooxygen ring vibrations. *Journal of Molecular Structure*.

[27] Alemi A., Khademinia S., Sertkol M. (2015). Part III: Lithium Metasilicate (Li2SiO3)—mild Condition Hydrothermal Synthesis, Characterization and Optical Properties. *International Nano Letters*.

[28] Gronkiewicz K., Majewski P., Wisniewska G., Pihut M., Loster B. W., Majewski S. (2009). Experimental research on the possibilities of maintaining thermal conditions within the limits of the physiological conditions during intraoral preparation of dental implants. *Journal of Physiology and Pharmacology*.

[29] White S. N., Suh P. S., Yu Z., Johnson R. (1997). Effect of fit adjustment on CEREC CAD-CAM veneers. *American Journal of Dentistry*.

[30] Van Zeghbroeck L. (2012). CAD/CAM treatment for the elderly - A case report. *Gerodontology*.

[31] Egawa M., Miura T., Kato T., Saito A., Yoshinari M. (2013). *In vitro* adherence of periodontopathic bacteria to zirconia and titanium surfaces. *Dental Materials*.

[32] Fasbinder D. J., Neiva G. F. (2016). Surface evaluation of polishing techniques for new resilient CAD/CAM restorative materials. *Journal of Esthetic and Restorative Dentistry*.

[33] Ribeiro M., Monteiro F. J., Ferraz M. P. (2012). Infection of orthopedic implants with emphasis on bacterial adhesion process and techniques used in studying bacterial-material interactions. *Biomatter*.

[34] Aktuğ S. L., Durdu S., Yalçın E., Çavuşoğlu K., Usta M. (2017). Bioactivity and biocompatibility of hydroxyapatite-based bioceramic coatings on zirconium by plasma electrolytic oxidation. *Materials Science and Engineering C: Materials for Biological Applications*.

[35] Valdez-Salas B., Beltrán-Partida E., Castillo-Uribe S. (2017). In Vitro Assessment of Early Bacterial Activity on Micro/Nanostructured Ti6Al4V Surfaces. *Molecules*.

[36] Zennaro C., Rastaldi M. P., Bakeine G. J. (2016). Ananoporous surface is essential for glomerular podocyte differentiation in three-dimensional culture. *International Journal of Nanomedicine*.

[37] Mehl C., Kern M., Schütte A.-M., Kadem L. F., Selhuber-Unkel C. (2016). Adhesion of living cells to abutment materials, dentin, and adhesive luting cement with different surface qualities. *Dental Materials*.

[38] Shenoy A., Shenoy N. (2010). Dental ceramics: an update. *Journal of Conservative Dentistry*.

[39] Roehling S., Astasov-Frauenhoffer M., Hauser-Gerspach I. (2017). In vitro biofilm formation on titanium and zirconia implant surfaces. *Journal of Periodontology*.

[40] Quirynen M., Marechal M., Busscher H. J. (1989). The Influence of Surface Free-energy on Planimetric Plaque Growth in Man. *Journal of Dental Research*.

[41] Rashid H. (2014). The effect of surface roughness on ceramics used in dentistry: a review of literature. *European Journal of Dentistry*.

[42] Tawakoli P. N., Attin T., Mohn D. (2016). Oral biofilm and caries-infiltrant interactions on enamel. *Journal of Dentistry*.

[43] Azarbal A., Azarbal M., Engelmeier R. L., Kunkel T. C. (2018). Marginal Fit Comparison of CAD/CAM Crowns Milled from Two Different Materials. *Journal of Prosthodontics*.

[44] Mota E. G., Smidt L. N., Fracasso L. M., Burnett L. H., Spohr A. M. (2017). The effect of milling and postmilling procedures on the surface roughness of CAD/CAM materials. *Journal of Esthetic and Restorative Dentistry*.

[45] Tabesh R., Dudley J. (2018). A Comparison of Marginal Gaps of All-Ceramic Crowns Constructed from Scanned Impressions and Models. *The International Journal of Prosthodontics*.

